# The association between the triglyceride–glucose index and prognosis in postoperative renal cell carcinoma patients: a retrospective cohort study

**DOI:** 10.3389/fendo.2024.1301703

**Published:** 2024-02-27

**Authors:** Guoliang Qin, Zhuang Sun, Yuxiang Jin, Xiangguo Ren, Zhaocun Zhang, Shuo Wang, Guanwen Zhou, Kun Huang, Haifeng Zhao, Xianzhou Jiang

**Affiliations:** ^1^ Department of Urology, Qilu Hospital of Shandong University, Jinan, China; ^2^ Department of Urology, Changle County People’s Hospital, Weifang, China

**Keywords:** renal cell carcinoma, prognosis, TyG index, OS, DFS, insulin resistance

## Abstract

**Background:**

Insulin resistance has been proven to be associated with renal cell carcinoma (RCC). However, the prognostic value of the triglyceride–glucose (TyG) index, as a marker for insulin resistance (IR), is still unclear. Therefore, we conducted research to explore the prognostic value and the predictive performance of the TyG index in postoperative RCC patients.

**Methods:**

A total of 651 postoperative RCC patients from January 2016 to June 2018 were enrolled in the final study. Their clinical and laboratory parameters were collected from medical records and through follow-up by phone. The triglyceride–glucose (TyG) index was calculated as follows: TyG = Ln[TG (mg/dl) × FBG (mg/dL)/2]. The overall survival (OS) and disease-free survival (DFS) were identified as the main outcomes.

**Results:**

The TyG index is an independent prognostic factor for OS (HR = 2.340, 95% CI = 1.506 to 3.64, *P* < 0.001) and DFS (HR = 2.027, 95% CI = 1.347 to 3.051, *P* < 0.001) in postoperative RCC patients. Kaplan–Meier survival curves of the different TyG index levels showed statistically significant differences in terms of OS and DFS (log-rank test, *P* < 0.0001). Furthermore, the TyG index was significantly associated with RCC risk factors.

**Conclusion:**

The TyG index is significantly associated with RCC survival. The mechanisms responsible for these results may contribute toward the improvement of RCC prognosis and immunotherapy efficacy and the development of new immunotherapeutic targets.

## 1 Introduction

Renal cell carcinoma (RCC) is the most common solid kidney lesion (1), and there has been an annual increase of 2% in RCC incidence worldwide over the past two decades (2). Surgical resection remains the only curative treatment for localized RCC (1). Although diagnostic and several treatment strategies have been developed, such as imaging technology, immunotherapy, and radiotherapy, the clinical outcomes remain unsatisfactory (3–6). Therefore, it is crucial to identify the potential prognostic factors for patient treatment selection and prognostic outcome improvement.

A growing body of evidence indicates that insulin resistance (IR), which is a major component of metabolic syndrome (MS) (7), may be associated with an increased risk as well as greater mortality rates for several types of cancers (7, 8). Metabolic syndrome comprises a cluster of metabolic abnormalities including hypertension, type 2 diabetes, obesity, and hyperlipidemia, and MS has been proven to be a risk factor for morbidity and poor prognosis of RCC (9–11). Moreover, previous studies showed that each component of MS is considered to have a close causal association with RCC (9, 11), and the pathophysiology seems to be largely attributable to IR (12, 13). Moreover, the visceral adiposity index (VAI), which is a predictor of IR, has been reported as a useful index to estimate the aggressiveness of RCC (14, 15). All evidence indicated that IR may play a crucial role in the progression of RCC and is a risk factor for poor outcomes.

The triglyceride–glucose (TyG) index has been evaluated as a reliable surrogate for IR for decades considering its consistency with the high insulin–glucose clamp test, the current gold standard for IR diagnosis (16–18). The association between the TyG index, as an insulin resistance marker and metabolic syndrome diagnostic factor, and the risk of cancers has been proven, and the results show that the TyG index was associated with the risk of RCC incidence (HR = 1.13, 95% CI = 1.07 to 1.20) (19, 20). However, only a few studies have explored the association between the TyG index and postoperative RCC outcomes.

Therefore, we aim to explore whether the TyG index can predict clinical outcomes in RCC patients and further explore the associations between the TyG index and other clinical prognostic characteristics of RCC.

## 2 Materials and methods

### 2.1 Study design and patients

This study was approved by the Ethics Review Committee of Qilu Hospital of Shandong University (Approval no. 2017067) and adhered to the Declaration of Helsinki. Considering that this is a retrospective study, we obtained verbal consent from all patients during the telephone follow-up.

We searched for patients hospitalized with renal tumors from January 2016 to June 2018, and 813 patients were included in the initial study cohort based on the inclusion criteria. The inclusion criteria were as follows: the first surgical treatment was performed at Qilu Hospital for a renal tumor, and there were accessible pathological reports for renal cell carcinoma; all clinical data were available and could be totally evaluable for the *post-hoc* analysis. Patients were followed up from March to August 2023, and 651 (80.1%) patients were included and provided verbal consent to participate in the study.

### 2.2 Variables and definitions

#### 2.2.1 Variables

All covariates included in the analysis were age, sex, smoking, drinking, metabolic disorders (hypertension, diabetes, and hyperlipidemia), serum total cholesterol (TC), triglyceride (TG), high-density lipoprotein cholesterol (HDL), low-density lipoprotein cholesterol (LDL), fasting plasma glucose (FPG), body mass index (BMI), TyG index, tumor size, T stage, N stage, M stage, clinical stage, renal cyst, pathological type and characteristics, operation (partial or radical), and surgical approach (laparotomy or laparoscopy). All results were collected from the most recent sequential examinations before surgery.

#### 2.2.2 Definition of variables and endpoints

BMI was defined as weight divided by height squared (kg/m^2^), and the cutoff values were defined by the Cooperative Meta-Analysis Group of China Obesity Task Force considering all the data in our study come from Qilu Hospital. The groups were as follows: underweight (BMI < 18.5 kg/m^2^), normal weight (BMI 18.5 to ≤23.9 kg/m^2^), overweight (BMI 24.0 to <28.0 kg/m^2^), and obese (BMI ≥ 28.0 kg/m^2^), and these groups were condensed to obese (≥28.0 kg/m^2^) versus non-obese (<28.0 kg/m2). Moreover, height and weight data were sourced from actual measurements during hospitalization rather than follow-up to reduce bias.

Metabolic disease was defined as patients with at least one of the following: diabetes, hypertension, or hyperlipidemia.

Diabetes was defined as a previous history of diabetes or fasting plasma glucose (FPG) greater than 7.0 mmol/L.

Hyperlipidemia was described as cholesterol >5.72 mmol/L, triglycerides >1.7 mmol/L, or HDL <1.0 mmol/L for male patients and <1.3 mmol/L for female patients.

The TyG index was calculated as follows: TyG = Ln[TG (mg/dl) × FBG (mg/dl)/2]. ROC analysis which took death from any cause as the endpoint based on the overall survival (OS) definition was conducted to calculate the Youden index. The most appropriate discriminatory cutoff value of the TyG index was 8.75 based on the maximum Youden index, and patients were classified into high (≥8.75) and low (<8.75) groups.

TNM stage was calculated using the European Association of Urology Guidelines on Renal Cell Carcinoma, which is updated in 2022.

Clinical endpoints included OS and disease-free survival (DFS). OS was defined as the interval between the day of surgery and the last follow-up or death from any cause. DFS was measured from the day of surgery and the first tumor recurrence or metastasis, last follow-up, or death of the subject for any reason.

### 2.3 Statistical analysis

Continuous variables are presented as mean ± standard deviation (SD), and categorical variables are presented as percentages.

We performed a receiver operating characteristic (ROC) curve analysis to test the sensitivity and specificity of the TyG index, and death from any cause based on the OS definition was considered as the endpoint. The Youden index is calculated as follows: Youden index = sensitivity + specificity − 1. The optimal cutoff value was selected based on the maximum Youden index. Kaplan–Meier survival curves and log-rank tests were used to compare the survival of different TyG index levels. Univariate and multivariate Cox proportional hazards models were used to show HRs and 95% confidence intervals (CIs). Furthermore, we conducted subgroup analysis and interaction analysis based on age, sex, BMI, diabetes, and hyperlipidemia to validate the efficacy of the TyG index in different populations. Pearson or Spearman correlation analysis was conducted to assess the association between the TyG index and RCC prognosis factors. The relationship between the TyG index and pathological characteristics was assessed by binary logistic regression and ordinal logistic regression. *P <*0.05 is considered statistically significant.

## 3 Results

### 3.1 Patient characteristics

A total of 651 patients were enrolled in the final study, with a median age of 56 years (range, 22 to 63), and 412 patients (63.3%) were men. All patients underwent an operation, i.e., partial nephrectomy (*n* = 261, 41%) and radical nephrectomy (*n* = 384, 59%). The baseline clinical characteristics are shown in **Table 1**. There were 227 (34.9%) patients with hypertension, 66 (10.1%) with diabetes, and 182 (27.9%) with hyperlipidemia. The optimal cutoff point of the TyG index was determined by the maximum Youden index of 8.75 stratifying all patients into low (<8.75, *N* = 442, 67.9%) and high (≥8.75, *N* = 209, 32.1%)groups. Non-obesity with BMI <28 kg/m^2^ was reported in 73.6% of the patients and obesity with BMI ≥28 kg/m^2^ in 26.5% of the patients.

**Table 1:** Baseline characteristics of RCC patients.

Characteristics	*N*
Age in years, median (range)	56 (22, 63)
Sex	
Female	239 (36.7%)
Male	412 (63.3%)
Smoking	147 (22.6%)
Drinking	152 (23.3%)
Hypertension	227 (34.9%)
Diabetes	66 (10.1%)
Hyperlipidemia	182 (27.9%)
Cholesterol (TC, mmol/L)	4.55 ± 0.95
HDL (mmol/L)	1.24 ± 0.28
LDL (mmol/L)	2.83 ± 0.81
FPG (mmol/L)	5.4 ± 1.2
Triglyceride (TG, mmol/L)	1.33 ± 0.68
TyG	7.25 ± 4.31
Low TyG (<8.75)	442 (67.9%)
High TyG (≥8.75)	209 (32.1%)
Size (cm)	5.2 ± 2.9
T status	NA = 45 (6.9%)
T1	469 (72%)
T2	103 (15.8%)
T3	33 (5.1%)
T4	1 (0.2%)
N1	7 (1.1%)
M1	9 (1.4%)
Stage (TNM stage)	NA = 45 (6.9%)
I	466 (71.6%)
II	95 (14.6%)
III	35 (5.4%)
IV	10 (1.5%)
BMI	NA = 109 (16.7%)
Non-obese (BMI <28 kg/m^2^)	398 (73.4%)
Obese (BMI ≥28 kg/m^2^)	144 (26.6%)
Surgical approach	
Laparotomy	27 (4.1%)
Laparoscopy	624 (95.8%)
Operation	
Partial	267 (41%)
Radical	384 (59%)
Pathological features	
Necrosis	46 (7.1%)
Bleeding	34 (5.2%)
Tumor thrombus	26 (4.0%)
Cystic degeneration	36 (5.5%)

Continuous variables are presented as mean ±  standard deviation (SD), and categorical variables are presented as percentages.

NA, not available; HDL, high-density lipoprotein; LDL, low-density lipoprotein; BMI, body mass index.

### 3.2 The TyG index and RCC outcomes

**Figure 1:**
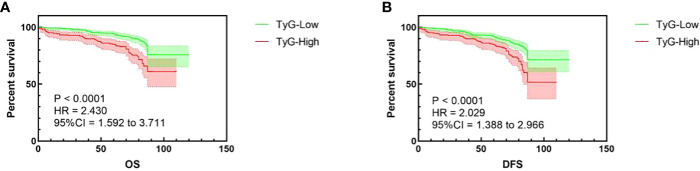
Kaplan–Meier curves in terms of OS **(A)** and DFS **(B)**.

The follow-up time was 62–89 months, and during the follow-up, 106 (16.2%) deaths from any cause, 18 (2.1%) recurrences, and 21 (3.2%) metastases were recorded. To explore the most optimal cutoff of the TyG index, we performed the ROC curve, and death from any cause based on the OS definition was considered as the endpoint (**Supplementary Figure S1**). According to the maximum Youden index (Youden index = sensitivity + specificity − 1), the optimal cutoff value for the TyG index was 8.75. Thus, the patients were subsequently divided into two different groups based on the optimal cutoff value: the high group (TyG ≥ 8.75) with 209 cases (32.1%) and the low group (METS-IR < 8.75) with 442 cases (67.9%) (**Table 1**). To show the different outcomes of different TyG index levels, we conducted the Kaplan–Meier survival curve. The curves indicated that a higher TyG index level led to worse RCC outcomes in terms of OS and DFS (**Figure 1**, log-rank test, *P* < 0.001).

**Figure 2:**
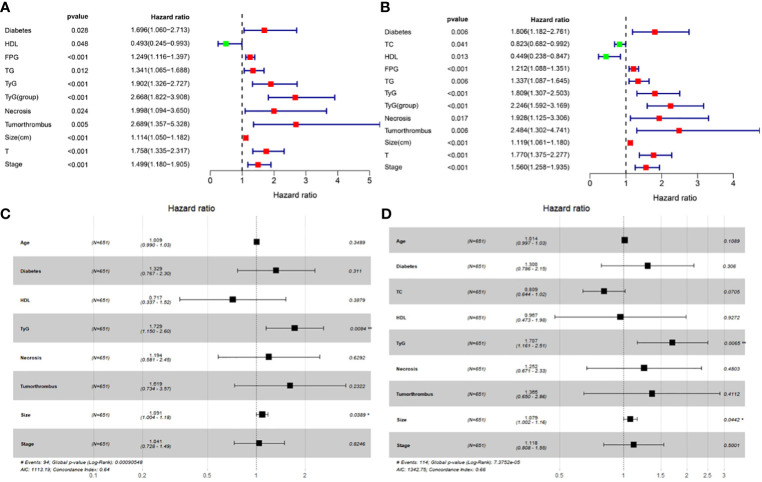
Univariate Cox proportional hazards regression analyses in RCC patients in terms of OS **(A)** and DFS **(B)**; multivariate Cox proportional hazards regression analyses according to OS **(C)** and DFS **(D)**.

Univariate and multivariate Cox proportional hazards regression analyses were performed to explore the association between factors and outcomes in terms of OS and DFS. In the univariate Cox analysis, we found several factors related to OS, including diabetes, HDL, FPG, TG, necrosis, tumor thrombus, tumor size, T status, and TNM stage (**Figure 2A**). The results showed that TyG was associated with OS as a continuous variable (HR = 1.902, 95% CI = 1.326 to 2.727, *P* < 0.001) and a categorical variable (HR = 2.668, 95% CI = 1.822 to 3.908, *P* < 0.001; **Figure 2A**). When DFS was considered as an outcome, we found that TyG still had statistical significance both as a continuous variable (HR = 1.809, 95% CI = 1.307 to 2.503, *P* < 0.001; **Figure 2B**) and a categorical variable (HR = 2.246, 95% CI = 1.592 to 3.169, *P* < 0.001; **Figure 2B**).

Multivariate Cox proportional hazards regression analysis showed that the TyG index was still significant after adjusting for confounders. The adjusted HR (95% CI) for the risk of death based on OS with per SD increase in the TyG index was 1.729 (1.150–2.60) (**Figure 2C**), and the risk increased by 70.7% (HR = 1.707, 95% CI = 1.161 to 2.51; **Figure 2D**) when adding recurrence and metastasis to the clinical endpoint in terms of DFS. Moreover, tumor size was also an independent risk factor for RCC survival.

### 3.3 Subgroup and interaction analyses

**Figure 3:**
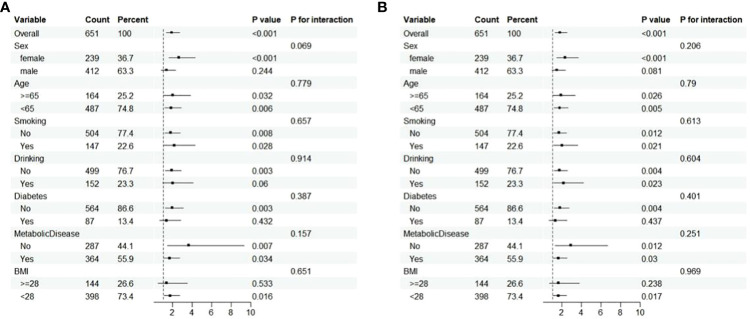
Subgroup analysis and interaction analysis based on OS **(A)** and DFS **(B)**.

The association between the TyG index and RCC survival was further examined in the subgroup analysis. Statistical significance was observed among women, people with no drinking history, patients without diabetes, non-obese patients with BMI <28 kg/m^2^, and all subgroups based on age, smoking, and metabolic disease for OS (**Figure 3A**). We also found statistical significance among women, patients without diabetes, non-obese patients with BMI <28 kg/m^2^, and all subgroups based on age, smoking, drinking, and metabolic disease for DFS (**Figure 3B**). Furthermore, no interaction was found between subgroup factors (all *P*-values >0.05; **Figure 3**).

### 3.4 Correlations between the TyG index and RCC prognosis factors

**Figure 4:**
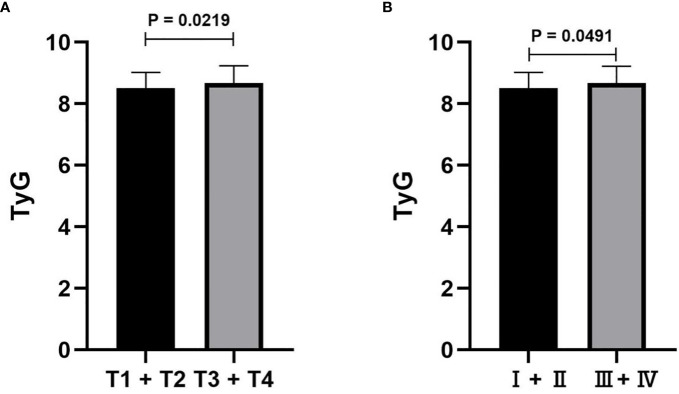
Correlations between the TyG index and TNM stage.

The association between the TyG index and RCC prognosis factors was examined, and the results are shown in **Table 2**. Spearman correlation analysis indicated that the TyG index was positively associated with cholesterol and LDL but negatively correlated with HDL (*P* < 0.05; **Table 2**). The logistic analysis demonstrated that TyG was related to adverse pathological features including bleeding (HR = 1.103, 95% CI = 1.037 to 1.174, *P* = 0.002; **Table 2**) and necrosis (HR = 1.067, 95% CI = 1.010 to 1.127, *P* = 0.022; **Table 2**) and higher Fuhrman grade (*P* = 0.004). T status and TNM stage did not show significance according to the parallel line assumption, and a *t*-test was used to study the correlation. The results showed that the TyG index was significantly related to T status (*P* = 0.0219; **Figure 4**) and TNM stage (*P* = 0.0491; **Figure 4**).

**Table 2:** Correlations between the TyG index and RCC risk factors.

Characteristics	Correlation coefficient (*r*)	*P*
Age	0.067	0.089
BMI	0.077	0.055
Cholesterol	0.202	**<0.001**
HDL	−0.181	**<0.001**
LDL	0.178	**<0.001**
Size (cm)	0.062	0.123
Necrosis	HR = 1.067 (1.010 to 1.127)	**0.022**
Tumor thrombus	HR = 0.948 (0.860 to 1.046)	0.289
Bleeding	HR = 1.103 (1.037 to 1.174)	**0.002**
N	HR = 1.050 (0.917 to 1.204)	0.479
M	HR = 0.973 (0.820 to 1.155)	0.755
Fuhrman	Parallel line assumption >0.05	**0.004**

Bold values means significant (P < 0.05).

## 4 Discussion

This was the first research to investigate the prognostic value of the TyG index in postoperative RCC patients. Some clinically significant results have been observed. The TyG index was related to worse outcomes for postoperative RCC patients and could independently predict OS and DFS. We also identified the cutoff value of the TyG index which could contribute to risk stratification. Furthermore, the significant association between the TyG index and RCC outcomes was mainly observed in specific subgroups including women, patients without diabetes, and non-obese patients with BMI <28 kg/m^2^. Moreover, the TyG index had a significant correlation with confirmed adverse prognostic factors. In summary, all the results indicated the prognostic value of the TyG index in postoperative RCC patients.

The association between IR and malignancy has been widely explored in the past decades (11). Previous epidemiological research into IR has focused on MS, especially on type 2 diabetes and hyperlipidemia (7). The presence of metabolic syndrome has been reported to lead to a greater risk and suggest a worse prognosis for many cancers including bladder cancer, prostate cancer, liver cancer, and renal cell cancer (11, 21). IR, which is a key component of MS, means that the individual’s cells and tissues become insensitive to the peptide hormone insulin (22, 23). A wealth of data has made it clear that a synergistic relationship exists between IR and cancer including RCC (22, 24, 25). The hyperinsulinemic–euglycemic clamp (HEC) is the gold standard in assessing the insulin sensitivity of peripheral tissues, and the homeostatic model assessment for insulin resistance (HOMA-IR) is a widely used method (26). However, HOMA-IR is expensive, difficult to operate, and affected by the use of insulin (27, 28). The TyG index, as a simple surrogate for IR, being comparable or even more predictive than HOMA-IR (16, 29, 30), had been reported to be a risk factor for RCC incidence (HR = 1.13, 95% CI = 1.07 to 1.20) (19, 20). However, no previous study focused on the prognostic value of the TyG index for postoperative RCC patients.

In the present study, we first revealed the predictive value of TyG in RCC patients, and these results were consistent with prior studies. All factors involved in the TyG index, including FPG and TG, had been reported to be risk factors for RCC survival (21, 24, 31, 32). Furthermore, our study indicated that the TyG index was associated with many RCC risk factors including TC, LDL, T status, stage, necrosis, and bleeding (1, 21, 24, 31, 32). Thus, the TyG index could be a complementary evaluation method to predict RCC survival.

In addition, research on the association between IR and RCC could provide a better understanding of the biological mechanisms of the prognostic value of the TyG index. Research on IR focuses on insulin-like growth factor (IGF), and IGF has been proposed as the key mechanism bridging insulin resistance and cancer (7, 21). IGF-1 is actually known to be related to poor survival and higher aggressiveness in RCC, and high IGF-1 level with established RCC may lead to impaired response to interleukin-2 (IL-2) therapy (33). Furthermore, the potent activity of IGF/IGF receptor 1 (IGF1R) inhibitors against RCC is demonstrated in basic research (33–36). Mammalian target of rapamycin (mTOR) inhibitors have led to the improvement of clinical outcomes in RCC, and a strong synergy is achieved combining IGF1R and mTOR inhibitors (35). Notably, insulin resistance treatment improves some indices of immune response, and the mechanisms accounting for immune deregulation in insulin resistance-related metabolic disorders may be related to metabolic reprogramming in RCC (7, 37, 38). These studies contribute toward the development of combination therapy utilizing metabolic target therapy and immunotherapy.

Obesity is usually the most common factor leading to insulin resistance. However, as observed in the subgroup analysis, the statistical association between the TyG index and RCC prognosis was only found among non-obese with BMI <28 kg/m² but not in obese patients with BMI ≥28 kg/m². Increasing evidence indicates the obesity paradox, which means that obesity leads to better survival in RCC patients, and the main causes are attributed to the less invasive nature of RCC in obese patients (39–41). Our results indicate that the obesity paradox may be related to the adaptation of obese individuals to insulin resistance, and abnormal insulin resistance in non-obese patients is more related to the prognosis of renal cancer and deserves more vigilance. The sex differences in the risk of cancers associated with IR have been reported before. A meta-analysis that included 38,940 cancer cases showed that female patients with metabolic syndrome had a higher risk of a number of cancers such as bladder and colorectal cancer than male patients (25), and it was also determined whether menopause is a key factor. This excess risk in female patients may be due to sex differences in body fat distribution and sex hormone secretion (42–44). These may explain the sex differences in the subgroup analysis. Moreover, diabetes and hyperlipidemia were also related to IR. According to follow-up data, we found that most patients with diabetes (87) clearly understand their condition and regularly take hypoglycemic drugs for treatment, while only 4 patients learned about their hyperlipidemia among 182 patients. Furthermore, the TyG index was reported to be more effective in detecting metabolic syndrome in non-diabetic patients (29). These reasons may partly explain the differences in the predictive power of the TyG index we observed in the subgroup analysis.

More importantly, the prognostic value of the TyG index may provide survival improvement for RCC patients. Many studies have revealed the inhibitory effect of metformin on RCC cells through induction of apoptosis and G0/G1 cell cycle arrest (45). Other studies report that fluvastatin could significantly inhibit tumor growth, invasion, angiogenesis, and metastasis of RCC cells *in vitro* (46). Recent studies have demonstrated that the long-term control status of blood glucose and lipid levels is more correlated with poor prognosis of tumors (21, 22, 26). Considering the relative paucity of laboratory and clinical research, further studies are warranted but reducing the TyG index may provide potential survival benefits for RCC patients.

Our study has some limitations which could not be avoided. First, this was a single-center retrospective study, potential bias was inevitable, and the cutoff value was only based on a single dataset. Second, the long-term TyG index change was not detected, and whether reducing the TyG index could enhance OS or DFS was not clear. Further multicenter, large-sample, prospective studies may strengthen our conclusion.

## 5 Conclusion

In conclusion, this study indicates that the TyG index could independently predict OS and DFS for postoperative RCC patients and the mechanisms that are responsible for these findings will potentially contribute toward the improvement of prognosis and the development of a therapeutic target. Therefore, the TyG index is a simple and reliable index for risk stratification and early intervention of postoperative RCC patients.

## Data availability statement

The original contributions presented in the study are included in the article/**Supplementary Material**. Further inquiries can be directed to the corresponding authors.

## Ethics statement

The studies involving humans were approved by Institutional Review Board (approval no. 2017067) of Qilu Hospital of Shandong University (Jinan, China). The studies were conducted in accordance with the local legislation and institutional requirements. As this was a retrospective cohort study and the follow-up was performed by phone, the ethics committee permitted verbal consent.

## Author contributions

GQ: Conceptualization, Formal analysis, Investigation, Methodology, Software, Visualization, Writing – original draft, Writing – review & editing. ZS: Conceptualization, Data curation, Investigation, Writing – original draft, Writing – review & editing. YJ: Conceptualization, Investigation, Writing – review & editing. XR: Conceptualization, Investigation, Writing – review & editing. ZZ: Conceptualization, Data curation, Investigation, Software, Writing – review & editing. SW: Conceptualization, Investigation, Writing – review & editing. GZ: Conceptualization, Investigation, Writing – review & editing. KH: Conceptualization, Investigation, Writing – review & editing. HZ: Conceptualization, Formal analysis, Investigation, Project administration, Supervision, Writing – review & editing. XJ: Conceptualization, Funding acquisition, Supervision, Writing – review & editing.
