# Supplementary figures and images for: The association between the triglyceride–glucose index and prognosis in postoperative renal cell carcinoma patients: a retrospective cohort study
Source: Front Endocrinol (Lausanne). 2024 Feb 27;15:1301703. doi: 10.3389/fendo.2024.1301703 (PMC10927751; doi:10.3389/fendo.2024.1301703)

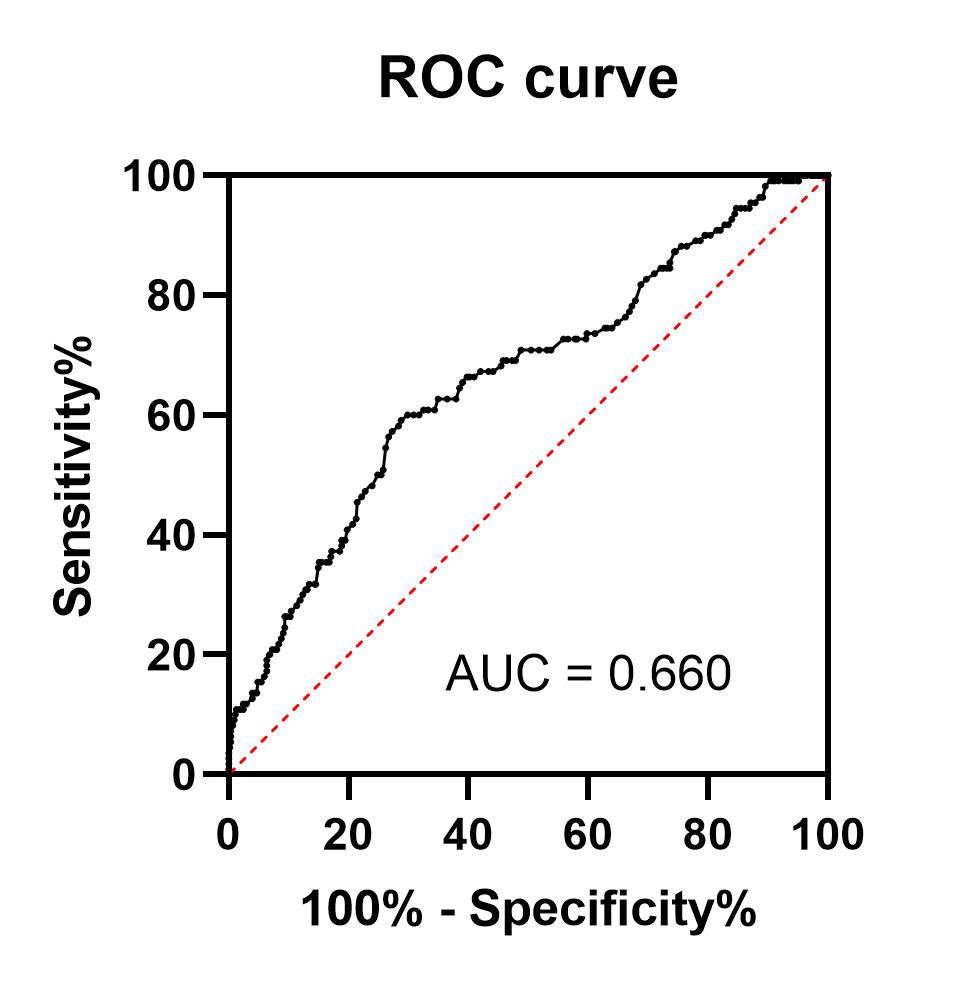

Supplement: Supplementary file 1 [file Image_1.jpeg]
